# A Clinical Risk Model to Predict Rapidly Progressive Interstitial Lung Disease Incidence in Dermatomyositis

**DOI:** 10.3389/fmed.2021.733599

**Published:** 2021-09-27

**Authors:** Yimin Li, Yuhui Li, Yuguang Wang, Lianjie Shi, Fuan Lin, Zongxue Zhang, Jingli Zhang, Yanying Liu, Xu Liu, Fangjingwei Xu, Xiaolin Sun

**Affiliations:** ^1^Beijing Key Laboratory for Rheumatism Mechanism and Immune Diagnosis, Department of Rheumatology and Immunology, Peking University People's Hospital, Beijing, China; ^2^Department of Respiratory, Beijing Hospital of Traditional Chinese Medicine, Beijing, China; ^3^Department of Rheumatology, Peking University International Hospital, Beijing, China; ^4^Department of Rheumatology, People's Hospital of Jianyang City, Jianyang, China; ^5^Department of Rheumatology, Hongqi Hospital of Mudanjiang Medical University, Mudanjiang, China; ^6^R&D Management Department, China National Biotec Group, Beijing, China

**Keywords:** dermatomyositis, rapidly progressive interstitial lung disease, risk prediction, risk marker, anti-MDA5 antibody

## Abstract

**Background:** Rapidly progressive interstitial lung disease (RP-ILD) is a fatal complication of dermatomyositis (DM) and clinically amyopathic DM (CADM). The objective of this study was to evaluate risk markers associated with RP-ILD incidence in patients with DM/CADM and to develop a RP-ILD risk prediction (RRP) model.

**Methods:** The clinical records of 229 patients with DM/CADM from Peking University People's Hospital, and 97 patients from four other independent clinical centers were retrospectively reviewed. Univariate and multivariate logistic regression analyses were performed to identify independent risk factors associated with later RP-ILD incidence to build a risk score model. The concordance index (C-index) and calibration curve were calculated to evaluate the predictive accuracy of the RRP model.

**Results:** A multiparametric RRP model was established based on weighted clinical features, including fever (yes, 5; no, 0), periungual erythema (yes, 6; no, 0), elevated CRP (yes, 5; no, 0), anti-MDA5 antibody (positive, 8; negative, 0), and anti-Ro-52 antibody (positive, 6; negative, 0). Patients were divided into three risk groups according to the RRP total score: low, 0–9; medium, 10–19; high, 20–30. The C-index and calibration curve of the RRP model showed a promising predictive accuracy on the incidence of RP-ILD.

**Conclusion:** The RRP model might promisingly predict the incidence of RP-ILD in DM/CADM patients to guide early individual treatment and further improve the prognosis of DM/CADM patients.

## Introduction

Dermatomyositis is an autoimmune disease characterized by skin and muscle damage caused by muscular involvement and frequent extramuscular symptoms such as Raynaud's phenomenon, arthritis, and interstitial lung disease (ILD) (1). Clinically amyopathic dermatomyositis (CADM) is a combination of hypomyopathic DM (HDM) and amyopathic DM (ADM), with characteristic skin-predominant lesions (2–5). ILD is one of the most severe complications of DM/CADM. Despite aggressive treatments, respiratory failure following rapidly progressive interstitial lung disease (RP-ILD) remains the main cause of death in more than 50% of DM/CADM patients (6–10). Consequently, the prediction and timely identification of RP-ILD symptom onset is vital for effective treatment during early disease development stages and might lead to improved prognosis with significantly reduced mortality rates in patients with DM/CADM (11–13).

A variety of studies have explored baseline parameters associated with RP-ILD in patients with DM/CADM but few practical quantitative methods were established and validated in clinical practice to predict the incidence of RP-ILD. Anti-melanoma differentiation-associated gene 5 (MDA5) antibody, lymphocytes in peripheral blood, C-reactive protein (CRP), skin ulceration, and ferritin were reported as predictive factors for disease onset and poor prognosis of RP-ILD (7, 8, 14, 15). However, previous studies were generally supported by limited cohort sizes or scattered case reports. Moreover, it seems reductive and inefficient to use a single clinical factor to predict the risk of an extremely heterogeneous disease as RP-ILD. In contrast, a holistic approach, based on multiple factors comprehensively evaluating personalized clinical characteristics, might provide a better predictive model for RP-ILD. Combining clinical and immunological factors might be valuable to evaluate disease severity, predict outcomes, and guide individualized treatment. Therefore, it is clinically significant to explore the RP-ILD-associated parameters at the onset of DM/CADM to establish a reliable early-stage RP-ILD risk prediction (RRP) model.

In this study, a practical score model to predict the incidence of RP-ILD was established through the identification and quantification of specific clinical parameters associated with later RP-ILD incidence in a patient cohort with DM/CADM. Moreover, this model was validated in different cohorts, aiming to guide personalized treatment during early RP-ILD development stages.

## Methods

### Patients

Demographic, clinical, and laboratory test data from 229 patients with DM/CADM admitted in Peking University People's Hospital from 2010 to 2019 was collected, and a retrospective analysis was performed to establish a risk score model to predict the RP-ILD incidence in the early stage of patients with DM/CADM. External validation was based on retrospective demographic, clinical, and laboratory test data from 97 patients with DM/CADM admitted in four other independent clinical centers (People's Hospital of Jianyang City, Peking University International Hospital, Beijing Hospital of Traditional Chinese Medicine, and Hongqi Hospital of Mudanjiang Medical University) from 2010 to 2019. Patients with idiopathic inflammatory myositis (IIM) were reviewed in this study. IIM was diagnosed according to criteria proposed by Bohan and Peter or 2017 European League Against Rheumatism and American College of Rheumatology (EULAR/ACR) (16, 17). The inclusion criteria include all the patients who had a definite DM or CADM diagnosis. DM was diagnosed according to the criteria of 2017 EULAR/ACR (17) and CADM was diagnosed according to criteria proposed by Sontheimer (5). By using incidence data of RP-ILD in DM/CADM of Peking University People's Hospital (33.6%, 77/229) and the other four independent clinical centers (9.3%, 9/97), the sample size was calculated in Power and Sample Size Free Calculators (http://www.powerandsamplesize.com/). According to the calculation results, the study sample size should include 41 patients in each cohort, with a one-sided α of 5%, and a power of 80%. In this study, the sample size collected was larger than 41. To improve the accuracy of the model, it was ensured that each group of samples was larger than 41. Finally, grouped by the time period, there were 165 cases in the development cohort from 2010 to 2015, 64 cases in the internal validation cohort from 2016 to 2019, and 97 cases in the external validation cohort from 2010 to 2019. This study was approved by the ethics committee of Peking University People's Hospital according to the declaration of Helsinki. The waiver of consent was agreed upon by the institutional ethics committee due to the retrospective nature of the study.

Patient exclusion criteria included recent acute infection, pulmonary infarction, presence of heart failure, history of neoplasm, other connective tissue diseases concomitantly, or insufficient demographic, clinical, and laboratory test data. ILD was diagnosed by the findings of high-resolution computed tomography (HRCT), according to the International Consensus Statement of Idiopathic Pulmonary Fibrosis of the American Thoracic Society (18) and defined as previously described (19). Chest HRCT patterns were classified into non-specific interstitial pneumonia (NSIP), organizing pneumonia (OP), NSIP combined with OP pattern. Pulmonary function tests (PFT) and Bronchoalveolar lavage (BAL) examinations were performed to evaluate ILD% predicted forced vital capacity (FVC), percent predicted diffusing lung capacity for carbon monoxide (DLco), and total lung capacity (TLC) at the initial diagnosis. Based on the radiological assessment of the chest HRCT results, RP-ILD was defined as a progressive deterioration of ILD in 3 months combining with rapidly progressive severe dyspnea and hypoxemia, requiring oxygen therapy or ventilator care (20, 21).

Demographic and clinical information including age at onset, gender, and initial symptoms, including fever, proximal muscle weakness, Gottron's sign/papules, skin ulceration, periungual erythema, and ILD were assessed. Periungual erythema was defined as erythematous rashes in perionychium, and erythema with accompanying changes including ulceration or black eschar. Gottron's sign/papules were defined as erythematous to violaceous papules, plaques, or macules (sign) over extensor surfaces of joints, which are sometimes scaly. Laboratory data included serological creatine kinase (CK), aspartate aminotransferase (AST), alanine transaminase (ALT), erythrocyte sedimentation rate (ESR), C-reactive protein(CRP), and lactate dehydrogenase (LDH). Myositis-specific autoantibodies (antigen panel, included Jo-1, PL-7, PL-12, EJ, OJ, KS, MDA5, NXP2, SAE, Mi-2, and TIF-1γ), and myositis-associated autoantibodies (antigen panel, included Ro-52, PM-Scl, and Ku) were screened in all patients by immunoblotting according to the instructions of the manufacturer (Euroimmun, Germany). Antinuclear antibodies (ANA) and rheumatoid factor (RF) were also recorded. All data were collected before initiating diagnosis.

### Statistical Analyses

Demographic and clinical characteristics of the development cohort and validation cohort were compared. Categoric variables were reported as counts (%) and compared using the χ^2^ test. Continuous variables were presented as the mean ± SD and compared by the ANOVA or the Kruskal–Wallis test. The least significant difference (LSD) of ANOVA and the chi-square test were used in a pair-wise *post-hoc* analysis. Logistic regression analysis and forward elimination process selection were used to explore independent risk factors with multivariate analyses. Results of the regression models were shown as the odds ratio (OR) with 95% confidence interval (95% CI). The performance of the RRP model was measured by the Harrel concordance index (C-index) and calibration curve in R version 3.6.1 (http://www.r-project.org/). The degree of agreement between the predicted probabilities with the actual outcomes numerically was measured by calibration curve, with a larger C-index value denoting better predictive accuracy. A value of *p* < 0.05 was considered statistically significant.

## Results

### Baseline Clinical Characteristics of the Patients

A total of 400 cases were included in this study, including 271 DM/CADM patients in Peking University People's Hospital and 129 DM/CADM patients in the other four independent hospitals. After screening for exclusion criteria, 229 patients in Peking University People's Hospital were included in the study for model development and internal validation, and 97 patients from the other four independent hospitals were included for the external validation cohort. The development cohort includes patients in the Peking University People's Hospital from 2010 to 2015 (*n* = 165), and the internal validation cohort includes the remaining patients from 2016 to 2019 (*n* = 64). The flowchart of patient inclusion in the Peking University People's Hospital is shown in Supplementary Figure 1, and the flowchart of external validation set is shown in Supplementary Figure 2.

Demographic, clinical, and laboratory characteristics of the development cohort and the internal validation cohort, the external validation cohort are summarized in Supplementary Table 1. The demographic, clinical, and laboratory manifestations of the three cohorts were compared with pair-wise *post-hoc* analyses in Supplementary Table 2. All patients underwent CT scans and none was diagnosed as RP-ILD at the baseline. At the outset, 84.8% (140/165) of the patients in the development cohort showed ILD onset on CT, but they were unable to be diagnosed as RP-ILD at that time. In the clinical practice, RP-ILD was diagnosed later, with the occurrence and development of the disease during 2 to 4 weeks of hospitalization, which was defined as a progressive deterioration of ILD in 3 months combining with rapidly progressive severe dyspnea and hypoxemia, requiring oxygen therapy or ventilator care (20, 21). Among 326 patients with DM/CADM, RP-ILD was found in 35.8% (59/165) of the developed cohort, 28.1% (18/64) of the internal validation cohort, 9.3% (9/97) of the external validation cohort, respectively. The incidence of RP-ILD did not differ among the three groups (*p* = 0.273, Supplementary Table 1). Based on the results of Supplementary Tables 1 and 2, it could be known that there was no difference between developed cohort and internal validation cohort in demographic, clinical, and laboratory factors, which was not like in pairs of internal validation cohort vs. external validation cohort and developed cohort vs. external validation cohort. Different features including Gottron's sign/papules, heliotrope rash, V sign, shawl sign, skin ulceration, fever, arthralgia, anti-aminoacyl-tRNA synthetase (ARS) antibodies, ANA, FVC% predicted, TLC% predicted, AST, ESR, and CRP, indicating that characteristics of patients were various in different hospitals. Detailed results were shown in Supplementary Tables 1, 2.

### Calculating the RRP Score

Univariate and multivariable logistic regression analyses were used to explore independent risk factors for RP-ILD incidence in DM/CADM patients. To accurately screen for risk factors, factors with *p* < 0.05 in the univariate analysis were included in multivariate analysis as covariates. Analyzed factors included mechanic's hands (yes vs. no), skin ulceration (yes vs. no), periungual erythema (yes vs. no), fever (yes vs. no), anti-MDA5 antibody (positive vs. negative), anti-Ro-52 antibody (positive vs. negative), and elevated CRP (yes vs. no) (Table 1). Multivariable logistic regression analysis indicated that fever, periungual erythema, anti-MDA5 antibody, anti-Ro-52 antibody, and elevated CRP were independent risk factors for RP-ILD in DM/CADM patients [fever: odds ratio (OR) = 3.407, 95% CI = 1.462-7.942, *p* = 0.005; periungual erythema: OR = 5.911, 95% CI = 1.886–18.531, *p* = 0.002; anti-MDA5 antibody: OR = 8.721, 95% CI = 3.147–24.166, *p* = 0.000; anti-Ro-52 antibody: OR = 6.244, 95% CI = 2.565–15.200, *p* = 0.000; elevated CRP: OR = 4.039, 95% CI = 1.679–9.716, *p* = 0.002; Table 1].

**Table 1:** Risk factors for RP-ILD according to the logistic regression model.

	**Univariate**			**Multivariate**		
	**OR**	**95% CI**	***P***	**OR**	**95% CI**	***P***
**Demographics**						
Age at onset, years	1.64	0.862–3.119	0.131			
Female (*n*, %)	1.397	0.662–2.947	0.38			
**Diagnosis**						
DM (*n*, %)	1.134	0.599–2.148	0.7			
CADM (*n*, %)	Reference					
**Clinical characteristics**						
Gottron's sign/papules (*n*, %)	1.358	0.597–3.089	0.466			
Mechanic's hands (*n*, %)	2.61	1.357–5.018	0.004			
Heliotrope rash (*n*, %)	1.068	0.562–2.03	0.841			
V sign (*n*, %)	1.101	0.58–2.088	0.769			
Shawl sign (*n*, %)	1.323	0.66–2.654	0.43			
Skin ulceration (*n*, %)	5.157	1.718–15.481	0.003			
Periungual erythema (*n*, %)	3.214	1.376–7.503	0.007	5.911	1.886–18.531	0.002
Myalgia (*n*, %)	1.289	0.554–2.997	0.555			
Myasthenia (*n*, %)	1.144	0.603–2.171	0.681			
Fever (*n*, %)	3.528	1.811–6.875	0.000	3.407	1.462–7.942	0.005
Dysphagia (*n*, %)	1.082	0.249–4.698	0.916			
Arthralgia (*n*, %)	1.379	0.728–2.612	0.325			
Raynaud's phenomenon (*n*, %)	2.361	0.609–9.158	0.214			
**Myositis-specific antibodies**						
Anti-ARS positivity	0.912	0.470–1.768	0.785			
Anti-Jo-1 positivity	0.542	0.243–1.21	0.135			
Anti-MDA5 positivity	6.804	3.031–15.274	0.000	8.721	3.147–34.166	0.000
Anti-Mi-2 positivity	0.496	0.100–2.470	0.392			
Anti-TIF1-γpositivity	0.378	0.079–1.812	0.224			
Anti-NXP2 positivity	0.244	0.029–2.032	0.192			
Anti-SAE positivity	1.825	0.250–13.3	0.553			
**Myositis-associated antibodies**						
Anti-Ro-52 positivity	3.611	1.833–7.115	0.000	6.244	2.565–15.200	0.000
Anti-PM/Scl-75/100 positivity	0.303	0.065–1.416	0.129			
Anti-Ku positivity	1.205	0.196–7.422	0.841			
RF	1.169	0.523–2.615	0.704			
ANA	1.270	0.599–2.694	0.533			
**HRCT**
OP	3.179	0.732–13.809	0.123			
NSIP	0.662	0.346–1.266	0.213			
OP+NSIP	2.093	0.957–4.577	0.064			
**Laboratory features**						
Elevated ALT (*n*, %)	1.913	0.995–3.678	0.052			
Elevated AST (*n*, %)	1.850	0.964–3.551	0.064			
Elevated LDH (*n*, %)	2.100	0.993–4.443	0.052			
Elevated CK (*n*, %)	0.598	0.286–1.252	0.173			
Elevated ESR (*n*, %)	1.126	0.592–2.140	0.718			
Elevated CRP (*n*, %)	3.223	1.63–6.373	0.001	4.039	1.679–9.716	0.002

*P-values were two-sided, and values of <0.05 were considered statistically significant. RP-ILD, rapidly progressive interstitial lung disease; DM, dermatomyositis; CADM: clinically amyopathic dermatomyositis; ARS include Jo-1, EJ, OJ, PL-7, PL-12, KS. ARS, aminoacyl-tRNA synthetase; MDA5, melanoma differentiation-associated 5; TIF-1γ, translation initiation factor-1γ; NXP2, nuclear matrix protein 2; SAE, small ubiquitin-like modifier enzyme; PM/Scl, polymyositis/scleroderma; ANA, antinuclear antibodies; RF, rheumatoid factor; HRCT, high-resolution computerized tomography; NSIP, nonspecific interstitial pneumonia; OP, organizing pneumonia; ALT, alanine transaminase; AST, aspartate aminotransferase; CK, creatine kinase; ESR: erythrocyte sedimentation rate; CRP, C-reactive protein; LDH, lactate dehydrogenase*.

The above five independent risk factors were then combined to establish an RRP model. Scores were based on five independent risk predictors weighted by regression coefficients and rounded to the nearest whole number (Table 2). Total risk scores were calculated by adding the weighted values of all prognostic variables. According to the RRP scores, patients were categorized into three risk groups: low-risk group, with RRP scores ranging from 0 to 9; medium-risk group, with RRP scores ranging from 10 to 19; high-risk group, with RRP scores ranging from 20 to 30.

**Table 2:** Calculation of the score for risk stratification in the development and validation cohorts.

	**OR (95%CI)**	***P*-value**	**β coefficient**	**Score**
Periungual erythema		0.002	1.777	
No	Reference group			0
Yes	5.911 (1.886–18.531)			6
Fever		0.005	1.226	
No	Reference group			0
Yes	3.407 (1.462–7.942)			5
Anti-MDA5 antibody		0.000	2.166	
Negative	Reference group			0
Positive	8.721 (3.147–34.166)			8
Anti-Ro-52 antibody		0.000	1.832	
Negative	Reference group			0
Positive	6.244 (2.565–15.200)			6
Elevated CRP		0.002	1.396	
No	Reference group			0
Yes	4.039 (1.679–9.716)			5

*Overall score: 0–9 low risk; 10–19 medium risk; 20–30 high risk. OR, odds ratio; CI, confidence intervals; MDA5, melanoma differentiation-associated 5; CRP, C-reactive protein*.

### Validation of the RRP Score System

**Figure 1:**
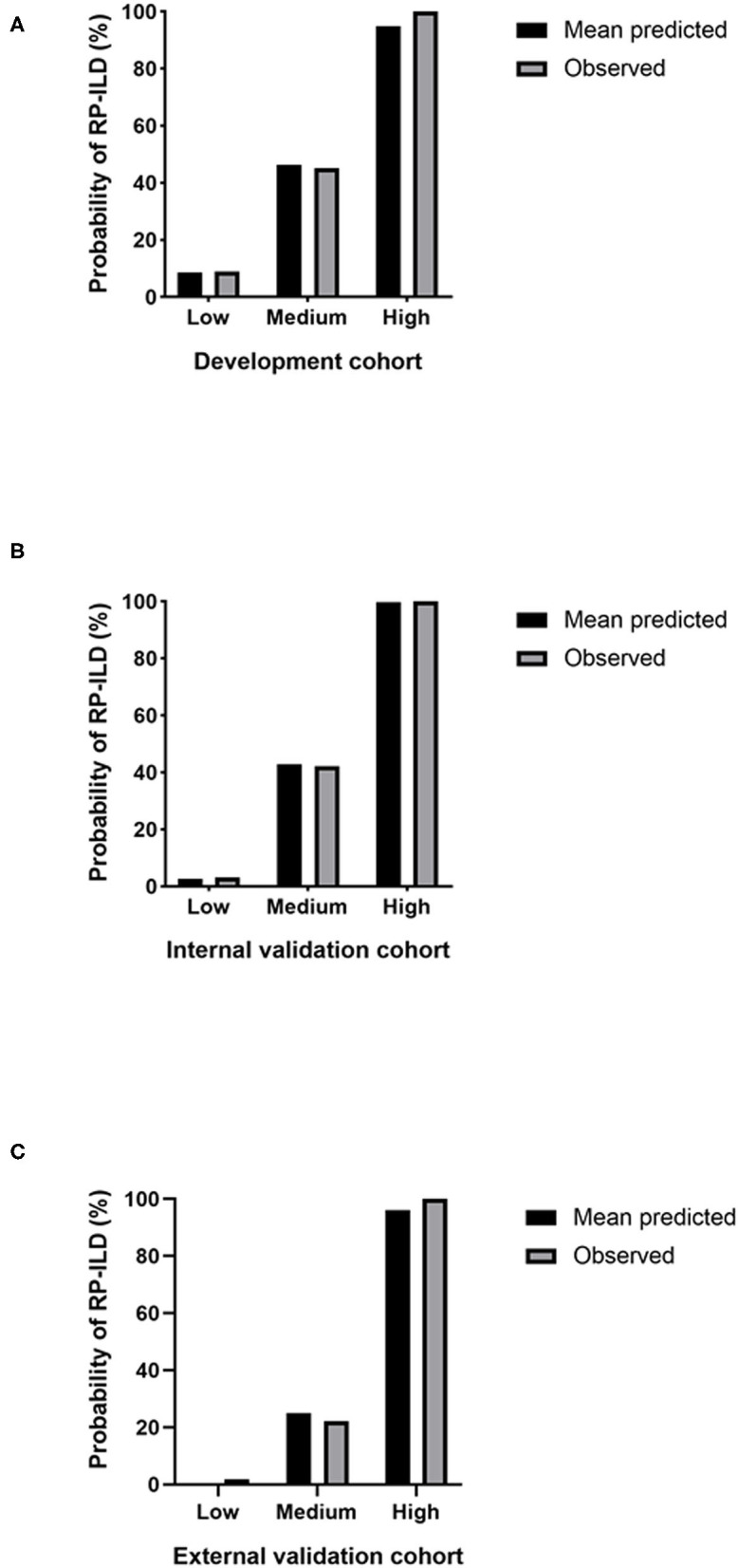
Predicted and observed rapidly progressive interstitial lung disease (RP-ILD) incidence in different risk groups. **(A)** The development cohort. **(B)** The internal validation cohort. **(C)** The external validation cohort. Low: low-risk; Medium: medium-risk; High: high-risk.

After classifying the development cohort according to the RRP scores, 40.6% of the patients were assigned to the low-risk group, 49.70% to the medium-risk group, and 9.69% to the high-risk group. The results of the internal validation cohort were similar: 50% of the patients were in the low-risk group, 40.63% in the medium-risk group, and 9.37% in the high-risk group. In the external validation cohort, 56.70% of the patients were assigned to the low-risk group, 37.11% to the medium-risk group, and 6.19% to the high-risk group (Table 3). The C-index for differences in RP-ILD incidence among risk groups from the development cohort was 0.849, with 95% CI 0.791 to 0.907. The mean predicted RP-ILD rates of the low-risk, medium-risk and high-risk groups were 8.61%, 46.41%, and 94.84%, respectively, while the observed rates were 8.96%, 45.12%, and 100%, respectively (Table 3, Figure 1A). Similarly, the C-index for differences in RP-ILD incidence rates among risk groups from the internal validation cohort was 0.928, with 95% CI 0.866 to 0.990 and the mean predicted RP-ILD rates of the low-risk, medium-risk, and high-risk groups were 2.7%, 42.87%, and 99.82%, with observed rates were 3.13%, 42.31%, and 100%, respectively (Table 3, Figure 1B). Finally, the C-index for differences in RP-ILD incidence rates among different risk groups from the external validation cohort was 0.948, with 95% CI 0.862 to 1.000 with mean predicted RP-ILD rates of the low-risk, medium-risk, and high-risk groups at 0.38%, 25.07%, and 96.14%, and observed rates were 1.82%, 22.22%, and 100%, respectively (Table 3, Figure 1C).

**Table 3:** Mean predicted and observed incidence of RP-ILD according to the risk category.

**Risk Category**	**DC (*****n*** **=** **165)**			**IVC (*****n*** **=** **64)**			**EVC (*****n*** **=** **97)**		
	**No (%)**	**Mean predicted incidence of RP-ILD (%)**	**Observed RP-ILD (%)**	**No (%)**	**Mean predicted incidence of RP-ILD (%)**	**Observed RP-ILD (%)**	**No (%)**	**Mean predicted incidence of RP-ILD (%)**	**Observed RP-ILD (%)**
Low	67 (40.61)	8.61	8.96	32 (50)	2.7	3.13	55 (56.70)	0.38	1.82
Medium	82 (49.70)	46.41	45.12	26 (40.63)	42.87	42.31	36 (37.11)	25.07	22.22
High	16 (9.69)	94.84	100	6 (9.37)	99.82	100	6 (6.19)	96.14	100
C-index	0.849			0.928			0.948		
(95%CI)	(0.791–0.907)			(0.866–0.990)			(0.862–1.000)		

*DC, development cohort; IVC, internal validation cohort; EVC, external validation cohort; CI, confidence intervals*.

**Figure 2:**
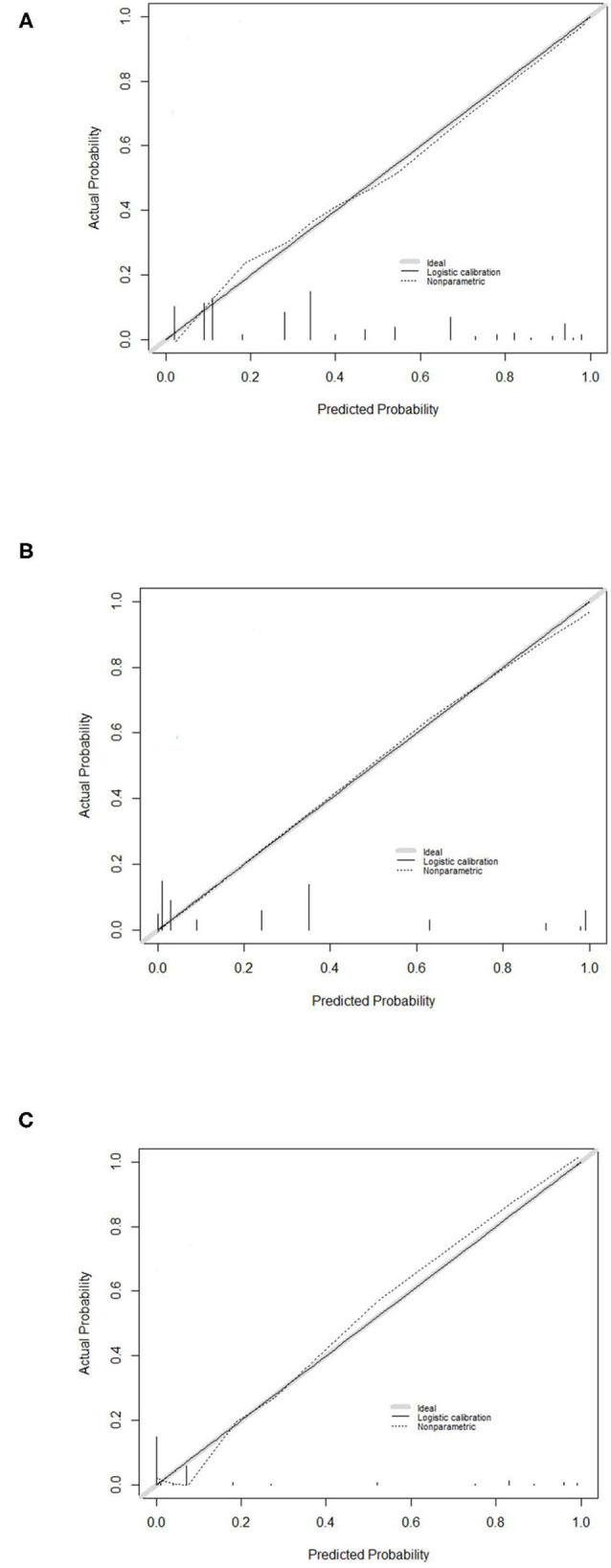
Calibration curve for the RP-ILD risk prediction (RRP) score system. Calibration curve for predicting RP-ILD in **(A)** the development cohort, **(B)** the internal validation cohort, and **(C)** the external validation cohort. RRP: RP-ILD risk prediction.

Moreover, the calibration plot based on bootstrap resampling validation demonstrated promising agreement between predicted and observed RP-ILD rates in the development cohort (Figure 2A), the internal validation cohort (Figure 2B), and the external validation cohort (Figure 2C).

## Discussion

Lung involvement is the most common reason for respiratory failure and death in DM/CADM. RP-ILD in DM patients is usually fatal within a few weeks or months (22). Therefore, predictive parameters for the onset of RP-ILD are important in the early treatment of DM/CADM patients. Previous studies mainly focused on short-term or small sample size studies to identify individual risk parameters of RP-ILD instead of exploring the multifactorial approach englobing the extensive clinical data from DM/CADM patients. Furthermore, none of the previous studies proposed a quantitative model to predict the risk of RP-ILD incidence in clinical practice. RP-ILD is an extremely heterogeneous disease. It is then likely, that multiple prognostic factors combined will better evaluate disease severity, predict outcomes, and guide individualized treatment than single-factor based models. Therefore, it was hypothesized that RP-ILD related risk parameters of DM/CADM could establish a dependable RRP model. In this study, an RRP model was built by combining independent predictive factors, including fever, periungual erythema, elevated CRP, anti-MDA5 antibody, and anti-Ro52 antibody. The early identification of RP-ILD progression is crucial for effective therapy, prognosis, and reduced mortality. In recent knowledge, this is the first score system available for clinical practice, which is able to predict the risk of RP-ILD incidence in DM/CADM patients during the early disease stages. Moreover, an independent external validation further confirmed the reliability of this model, with high accuracy in patients with a high risk of RP-ILD incidence.

Predictors of poor outcome during DM-ILD have been previously reported (23–25), however, fewer studies assessed the prognostic factors for myositis-associated RP-ILD. This study confirmed many single clinical and laboratory prognostic factors previously reported, such as periungual erythema, fever, and CRP, as risk factors for RP-ILD in myositis (8, 15, 26–28). However, the majority of predictive factors for ILD (8, 15, 26, 27, 29), e.g., skin ulceration and hands of a mechanic, which have been previously identified by univariate analysis, were not classified as independent risk factors by multivariate analysis in the study.

A dose-dependent relationship between anti-MDA5 antibody and RP-ILD has been recently reported, unveiling anti-MDA5 antibodies as a significant risk factor for poor prognosis (30). Similarly, patients with anti-Ro-52 antibodies during juvenile myositis are more likely to develop severe ILD with a poor prognosis (31). Consistent with these reports, this study also demonstrated that anti-MDA5 antibody and anti-Ro-52 antibody were both specific biomarkers for DM/CADM-associated RP-ILD (8, 32).

Based on the results in Supplementary Tables 1 and 2, it was found that the characteristics of patients in different hospitals were not the same. Therefore, there was a need to establish an evaluation system with a universal applicability in different hospitals. In the RRP score model of the study, patients with a score between 0 and 9 are considered as low-risk subgroups for the development of RP-ILD, while scores between 10 and 19 and 20 or more are at medium-risk and high-risk of RP-ILD development, respectively. The results of summarized calibration and discrimination for the RRP model in three cohorts all presented a high predictive accuracy for estimating the risk of RP-ILD supported in clinical settings. Therefore, researchers of the study thought that this model has general applicability, which still needs to be proved in the future.

This study presented several limitations. First, this study was a retrospective study, suboptimal compared to the prospective study and included hospitalized patients only. Therefore, it might have shown biases in patient selection. Second, the prediction model was only tested in a Chinese population. The generalizability of this model to other ethnicities needs to be validated in future studies. Third, the validation cohort has a limited sample size because of the rarity of the disease; this model needs to be further validated by more multi-center studies with enlarged patient cohorts. Fourth, this risk score was mainly based on clinical characteristics. Although several studies have reported that some serum biomarkers, including ferritin, KL-6, and IL-18, were related to RP-ILD and might be used as potential biomarkers for predicting disease severity and prognosis (10, 33); the researchers of the study were unable to include these markers in the model due to the lack of these data in the retrospective study, whether those markers could be used as a reliable alternative biomarker needs further validation.

## Conclusions

The first RRP score model capable of predicting the risk of RP-ILD incidence during early disease stages was provided. It is expected to be a valuable clinical tool that could guide early personalized treatment, improve prognosis, and reduce morality for DM/CADM patients.

## Data Availability Statement

The raw data supporting the conclusions of this article will be made available by the authors, without undue reservation.

## Ethics Statement

This study was approved by the Ethic Committee of Peking University People's Hospital according to the declaration of Helsinki. Waiver of consent was agreed upon by the institutional ethics committee due to the retrospective nature of the study.

## Author Contributions

XS, YHL, and FX contributed to the conception or design of the study. YML contributed to the analysis of the data. YW, LS, FL, ZZ, JZ, YYL, and XL contributed to the collection of the data. All authors were involved in the interpretation of the data and reviewed and approved the letter's content before submission.

## Funding

This work was supported by National Natural Science Foundation of China (81971520 and 81801617), Peking University People's Hospital Research and Development Funds (RDX2019-03, RDX2020-03).

## Conflict of Interest

FX was employed by the company China National Biotec Group. The remaining authors declare that the research was conducted in the absence of any commercial or financial relationships that could be construed as a potential conflict of interest.

## Publisher's Note

All claims expressed in this article are solely those of the authors and do not necessarily represent those of their affiliated organizations, or those of the publisher, the editors and the reviewers. Any product that may be evaluated in this article, or claim that may be made by its manufacturer, is not guaranteed or endorsed by the publisher.
