# Supplementary figures and images for: A Clinical Risk Model to Predict Rapidly Progressive Interstitial Lung Disease Incidence in Dermatomyositis
Source: Front Med (Lausanne). 2021 Sep 27;8:733599. doi: 10.3389/fmed.2021.733599 (PMC8502922; doi:10.3389/fmed.2021.733599)

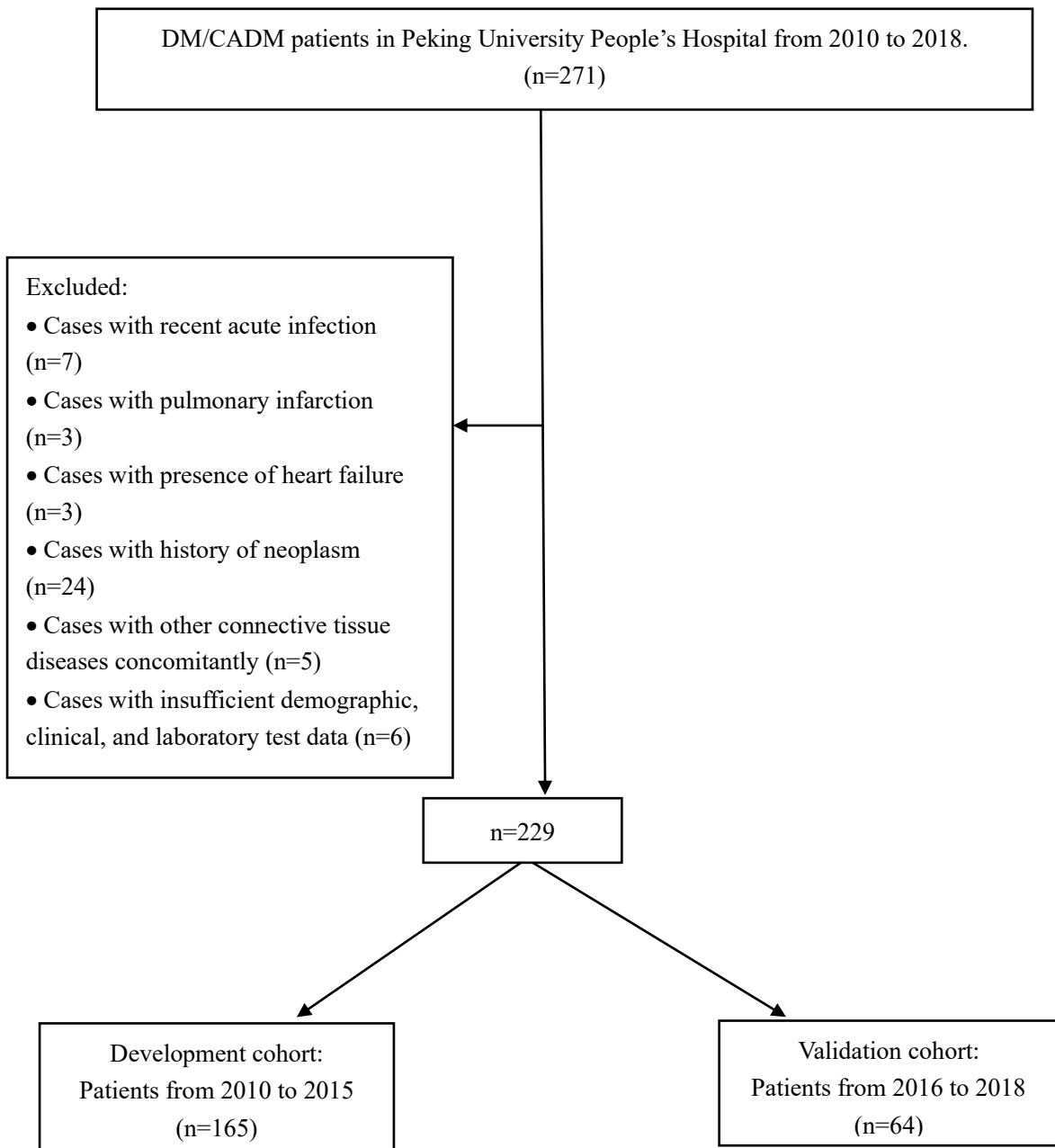

Supplement: Supplementary file 1 [file Image_1.pdf]
