# Supplementary material for: A Clinical Risk Model to Predict Rapidly Progressive Interstitial Lung Disease Incidence in Dermatomyositis
Source: Front Med (Lausanne). 2021 Sep 27;8:733599. doi: 10.3389/fmed.2021.733599 (PMC8502922; doi:10.3389/fmed.2021.733599)
Supplement: Supplementary file 2 [file Image_2.pdf]

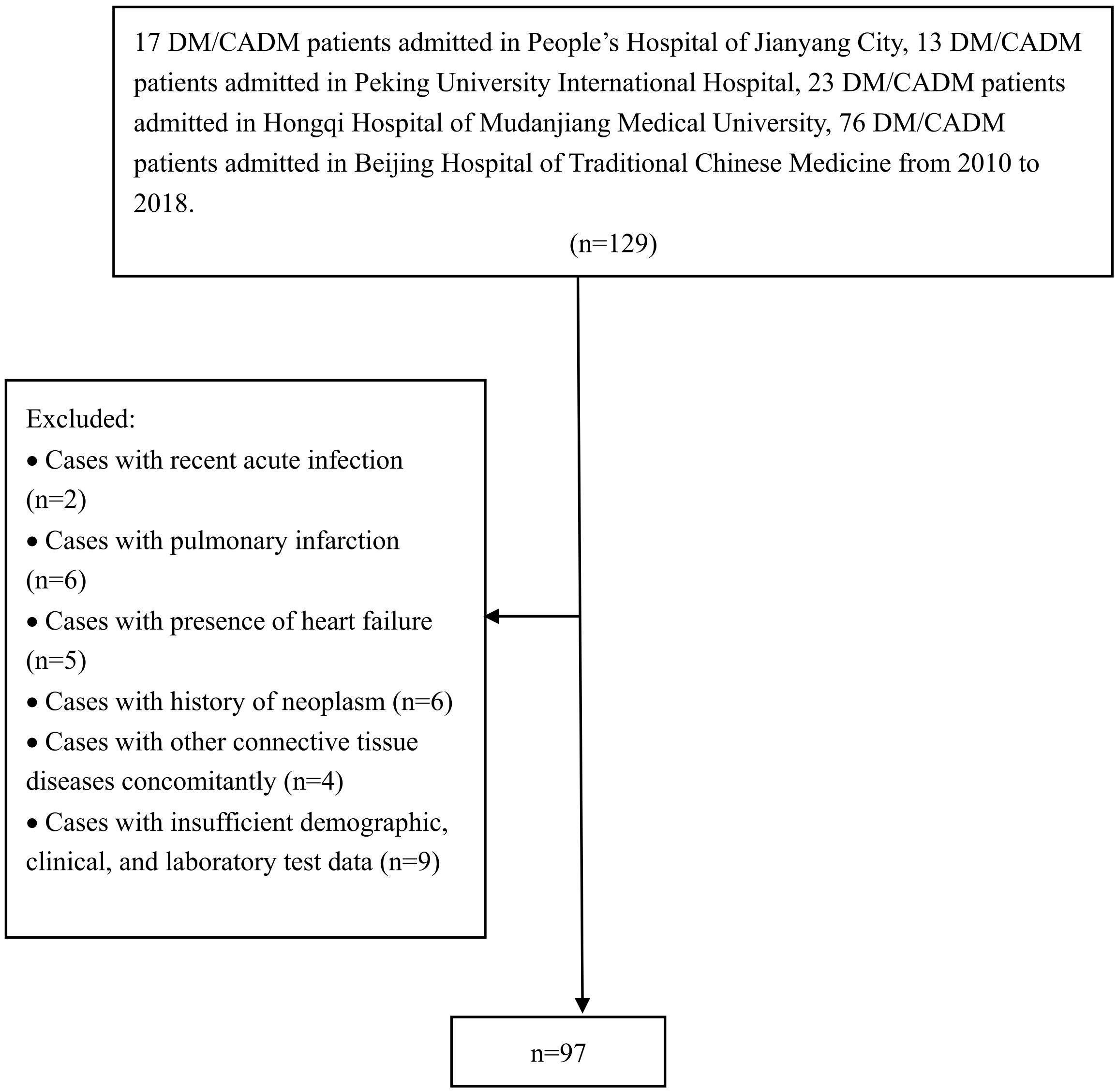
17 DM/CADM patients admitted in People's Hospital of Jianyang City, 13 DM/CADM patients admitted in Peking University International Hospital, 23 DM/CADM patients admitted in Hongqi Hospital of Mudanjiang Medical University, 76 DM/CADM patients admitted in Beijing Hospital of Traditional Chinese Medicine from 2010 to 2018.
(n=129)
Excluded:
• Cases with recent acute infection (n=2)
• Cases with pulmonary infarction (n=6)
• Cases with presence of heart failure (n=5)
• Cases with history of neoplasm (n=6)
• Cases with other connective tissue diseases concomitantly (n=4)
• Cases with insufficient demographic, clinical, and laboratory test data (n=9)
n=97
