# Supplementary material for: A Clinical Risk Model to Predict Rapidly Progressive Interstitial Lung Disease Incidence in Dermatomyositis
Source: Front Med (Lausanne). 2021 Sep 27;8:733599. doi: 10.3389/fmed.2021.733599 (PMC8502922; doi:10.3389/fmed.2021.733599)
Supplement: Supplementary file 4 [file Table_2.docx]

**Supplementary Table S2. Pair-wise post-hoc analysis for clinical and laboratory descriptive statistics in the development cohort, the internal validation cohort and the external validation cohort**

|  | DC (n =165) | IVC (n =64) | EVC (n=97) | *P*  DC vs IVC | *P*  DC vs EVC | *P*  IVC vs EVC |
| --- | --- | --- | --- | --- | --- | --- |
| Demographics |  |  |  |  |  |  |
| Age at onset, years | 51.1±12.9 | 49.0±15.5 | 53.8±11.4 | 0.274 | 0.104 | 0.022 |
| Female (n, %) | 122 (73.9) | 46 (71.9) | 76 (78.3) | 0.751 | 0.422 | 0.348 |
| Diagnosis |  |  |  | 0.685 | 0.322 | 0.245 |
| DM (n, %) | 75 (45.5) | 31(48.4) | 38(39.2) |  |  |  |
| CADM (n, %) | 90 (54.5) | 33(51.6) | 59(60.8) |  |  |  |
| Clinical characteristics |  |  |  |  |  |  |
| RP-ILD (n, %) | 59(35.8) | 18(28.1) | 9(9.3) | 0.273 | 0.000 | 0.002 |
| Gottron’s sign/papules (n, %) | 132(80.0) | 48(75.0) | 23(23.7) | 0.517 | 0.000 | 0.000 |
| Mechanic's hands (n, %) | 73(44.2) | 24(37.5) | 33(34.0) | 0.354 | 0.104 | 0.651 |
| Heliotrope rash (n, %) | 71(43.0) | 33(51.6) | 18(18.6) | 0.245 | 0.000 | 0.000 |
| V sign (n, %) | 73(44.2) | 33(51.6) | 11(11.3) | 0.319 | 0.000 | 0.000 |
| Shawl sign (n, %) | 47(28.5) | 26 (40.6) | 8 (8.2) | 0.077 | 0.000 | 0.000 |
| Skin ulceration (n, %) | 17(10.3) | 3 (4.7) | 2 (2.1) | 0.276 | 0.013 | 0.347 |
| Periungual erythema (n, %) | 27(16.4) | 7 (10.9) | 9(9.3) | 0.300 | 0.108 | 0.731 |
| Subcutaneous calcinosis (n, %) | 5(3.0) | 2(3.1) | 0(0) | 1.000 | 0.083 | 0.080 |
| Myalgia (n, %) | 27(16.4) | 12(18.8) | 27(27.8) | 0.666 | 0.027 | 0.188 |
| Myasthenia (n, %) | 72(43.6) | 31(48.4) | 37(38.1) | 0.512 | 0.384 | 0.196 |
| Fever (n, %) | 66(40.0) | 20(31.3) | 12(12.4) | 0.220 | 0.000 | 0.003 |
| Dysphagia (n, %) | 8(4.8) | 2(3.1) | 8(8.2) | 0.832 | 0.267 | 0.188 |
| Arthralgia (n, %) | 81(49.1) | 24(37.5) | 16(16.5) | 0.114 | 0.000 | 0.003 |
| Raynaud's phenomenon | 9(5.5) | 3(4.7) | 7(7.2) | 1.000 | 0.565 | 0.515 |
| Myositis-specific antibodies |  |  |  |  |  |  |
| Anti-ARS positivity | 61(37.0) | 21(32.8) | 77(79.4) | 0.556 | 0.000 | 0.000 |
| Anti-Jo-1 positivity | 39(23.6) | 10(15.6) | 21(21.6) | 0.185 | 0.712 | 0.343 |
| Anti-MDA5 positivity | 37(22.4) | 11(17.2) | 15(15.5) | 0.382 | 0.173 | 0.771 |
| Anti-Mi-2 positivity | 9(5.5) | 5(7.8) | 6(6.2) | 0.504 | 0.806 | 0.689 |
| Anti-TIF1-γpositivity | 11(6.7) | 8(12.5) | 3(3.1) | 0.151 | 0.214 | 0.021 |
| Anti-NXP2 positivity | 8(4.8) | 5 (7.8) | 1 (1.0) | 0.384 | 0.101 | 0.026 |
| Anti-SAE positivity | 4(2.4) | 3 (4.7) | 1 (1.0) | 0.642 | 0.426 | 0.145 |
| Myositis-associated antibodies |  |  |  |  |  |  |
| Anti-Ro-52 positivity | 82(49.7) | 29(45.3) | 45(46.4) | 0.551 | 0.605 | 0.893 |
| Anti-PM/Scl-75/100 positivity | 13 (7.9) | 5 (7.8) | 4 (4.1) | 0.987 | 0.233 | 0.319 |
| Anti-Ku positivity | 5 (3.0) | 5 (7.8) | 2 (2.1) | 0.112 | 0.639 | 0.080 |
| RF | 31 (18.8) | 14 (21.9) | 19 (19.6) | 0.598 | 0.874 | 0.725 |
| ANA | 124 (75.2) | 51 (79.7) | 51 (52.6) | 0.468 | 0.000 | 0.000 |
| HRCT |  |  |  |  |  |  |
| OP | 8 (4.8) | 1 (1.6) | 9(9.3) | 0.442 | 0.160 | 0.099 |
| NSIP | 100(60.6) | 34(53.1) | 49(50.5) | 0.302 | 0.111 | 0.746 |
| OP+NSIP | 32(19.4) | 7(10.9) | 19(19.6) | 0.127 | 0.970 | 0.144 |
| Pulmonary function tests^a^ |  |  |  |  |  |  |
| FVC % predicted | 75.9±18.6 | 78.7±21.6 | 48.8±22.7 | 0.528 | 0.000 | 0.000 |
| DLco % predicted | 63.2±20.5 | 60.4±22.8 | 65.7±27.8 | 0.579 | 0.539 | 0.322 |
| TLC % predicted | 80.3±17.4 | 83.2±21.0 | 62.2±26.4 | 0.546 | 0.000 | 0.000 |
| Bronchoalveolar lavage^b^ |  |  |  |  |  |  |
| Total cell number, 10^5^/ml | 0.32±0.36 | 0.27±0.22 | 0.67±0.61 | 0.539 | 0.081 | 0.057 |
| Macrophage, % | 51.2±24.5 | 54.2±20.5 | 52.8±25.3 | 0.593 | 0.908 | 0.923 |
| Lymphocyte, % | 37.7±24.4 | 28.5±16.5 | 36.8±30.5 | 0.096 | 0.949 | 0.555 |
| Neutrophil, % | 9.8±14.8 | 15.4±19.0 | 6.0±4.4 | 0.142 | 0.681 | 0.333 |
| Eosinophil, % | 1.2±2.9 | 1.8±2.4 | 1.3±1.5 | 0.63 | 0.925 | 0.783 |
| Laboratory features |  |  |  |  |  |  |
| Elevated ALT (n, %) | 62(37.6) | 27(42.2) | 32(33.0) | 0.521 | 0.455 | 0.236 |
| Elevated AST (n, %) | 87(53.0) | 39(60.9) | 37(38.1) | 0.282 | 0.020 | 0.005 |
| Elevated LDH (n, %) | 88(53. 3) | 40(62.5) | 64(66.0) | 0.210 | 0.045 | 0.651 |
| Elevated CK (n, %) | 47(28.5) | 23(35.9) | 38(39.2) | 0.272 | 0.074 | 0.679 |
| Elevated ESR (n, %) | 92(55.8) | 42(65.6) | 34(35.1) | 0.174 | 0.001 | 0.000 |
| Elevated CRP (n, %) | 116(70.3) | 46(71.9) | 13(17.1) | 0.814 | 0.000 | 0.000 |

^a^ Number of subjects with DC, n=82, Number of subjects with IVC, n=29, Number of subjects with EVC, n=65, ^b^ Number of subjects with DC, n=73, Number of subjects with IVC, n=22, Number of subjects with EVC, n=3. The LSD of analysis of variance and chi square test were used in a pair-wise post-hoc analysis. *P* values were two-sided, and values of < 0.05 were considered statistically significant. DC: development cohort; IVC: internal validation cohort; EVC: external validation cohort; RP-ILD: rapidly progressive interstitial lung disease; DM: dermatomyositis; CADM: clinically amyopathic dermatomyositis; ARS include Jo-1, EJ, OJ, PL-7, PL-12, KS. ARS: aminoacyl-tRNA synthetase; MDA5: melanoma differentiation-associated 5; TIF-1γ: translation initiation factor-1γ; NXP2: nuclear matrix protein 2; SAE: small ubiquitin-like modifier enzyme; PM/Scl: polymyositis/scleroderma; ANA: antinuclear antibodies; RF: rheumatoid factor; HRCT, high resolution computerized tomography; NSIP, nonspecific interstitial pneumonia; OP, organizing pneumonia; FVC: forced vital capacity; DLCO, diffusion capacity for carbon monoxide; TLC, total lung capacity; ALT: alanine transaminase; AST: aspartate aminotransferase; CK: creatine kinase; ESR: erythrocyte sedimentation rate; CRP: C-reactive protein; LDH: lactate dehydrogenase.
